# Psychiatric morbidity amongst adolescents in a Nigerian juvenile correctional facility

**DOI:** 10.4102/sajpsychiatry.v27i0.1590

**Published:** 2021-08-16

**Authors:** Amudalat T. Kuranga, Abdullahi D. Yussuf

**Affiliations:** 1 Department of Behavioural Science, University of Ilorin Teaching Hospital, Kwara State, Nigeria

**Keywords:** psychiatric morbidity, juveniles, juvenile justice system, adolescents, Borstal Institution, Nigeria

## Abstract

**Background:**

The high occurrence of psychiatric disorders amongst adolescents within the Juvenile Justice System (JJS) has been confirmed. Most of the available data are from developed countries and some of them focus on just a single psychiatric disorder which may not be representative of the situation in low-income countries, hence the need for more studies in developing countries, including Nigeria.

**Aim:**

The study aimed to determine the prevalence of psychiatric disorders amongst adolescent residents of a correctional facility.

**Setting:**

The study was carried out at a Borstal Institution in North-Central Nigeria.

**Methods:**

A descriptive cross-sectional study design was used. One hundred and twenty adolescents were assessed using the socio-demographic pro forma questionnaire designed by the researcher and the Kiddies Schedule for Affective Disorders and Schizophrenia (KSADS-PL). Data were analysed using EPI-INFO 4.06 d version 6.04 software.

**Results:**

A total of 62.5% of the male respondents were older than 15 years. The percentage of respondents with a psychiatric disorder was 82.5%. The rate of psychiatric disorders was high with disruptive behaviour disorders being the most common at 40.8%, others were substance use disorders (15.8%), anxiety disorders (14.2%), psychosis (6.7%) and mood disorders (5%).

**Conclusion:**

This study has established a high prevalence rate of psychiatric disorders amongst incarcerated adolescents. This is in line with the findings of numerous studies worldwide. This study has identified the need to increase awareness and knowledge about the high morbidity of mental disorders in growing juvenile detainee populations. This will allow early identification of adolescents at risk of psychiatric disorders and ensure efficient resource distribution of both JJS service and mental healthcare. Effective and appropriate interventions have shown to improve overall health, quality of life and reduce the rate of recidivism amongst incarcerated juveniles.

## Introduction

Juvenile crime is a perennial public concern.^1^ It is one of the most pressing social problems with detrimental emotional, physical and economic effects felt through the communities in which it occurs.^2^

Adolescents in the juvenile correctional system are a high-risk population who, in many cases, have unmet physical, developmental and mental health needs, which occur at a higher rate than in the general population.^3^ The increasing number of adolescent offenders in the Juvenile Justice System (JJS) with psychiatric disorders is a major public health problem.^4^ Several studies have shown that adolescents with substance abuse or mental health disorders consistently have higher offending and reoffending rates, poor prognosis of mental health problems,^5^ increased likelihood to perpetrate or experience violence in intimate relationships and psychosocial difficulties in adulthood.^6^

Vander et al.^7^ documented that adolescents enrolled in a public mental health system had three times as many police referrals to the JJS as those in the general child population.

Several studies have reported a high prevalence rate of psychiatric disorders amongst adolescents involved with the JJS worldwide, including Africa,^8,9,10^ and it has been documented that almost two-thirds of youth in juvenile justice detention meet the criteria for one or more mental disorders, thereby posing a challenge to both the Juvenile Justice and mental health system.^11^ Findings from a study of institutionalised adolescents in Sweden showed that 73% of them had at least one psychiatric disorder, 48% had attention deficit hyperactivity disorder, 17% autism spectrum disorder and 10% had intellectual disability.^12^ A systematic literature review by Collins et al.^13^ showed the mean prevalence of any psychiatric disorder to be (69.9%) with conduct disorder occurring most frequently (46.4%), followed by substance use disorder (45.1%), oppositional defiant disorder (ODD) (19.8%) and attention deficit hyperactivity disorder (13.5%). High degrees of comorbidity of mental disorders have also been reported amongst juvenile detainees. Abram et al.^4^ showed that 56.5% of girls and 45.9% of boys in the Cook County juvenile temporary detention centre in Illinois, United States of America (USA) fulfiled the criteria for two or more disorders. A study performed in a juvenile court in Kenya reported a crude psychiatric morbidity rate of 44.4%, of which 45% had conduct disorder, 20% had mixed disorders of conduct and emotion, 20% had emotional disorders with onset specific to childhood, 12.5% had mood disorders and 2.5% had hyperkinetic disorders.^14^

Given the growth of the juvenile detainee population, epidemiologic data on their psychiatric disorders are becoming increasingly important,^15^ yet there are few empirical studies,^15^ especially in Africa. A recent systematic scoping review concluded that a modest number of studies have been conducted on psychiatric morbidity amongst adolescents involved with the JJS in sub-Saharan Africa, but there are still significant research gaps that need to be filled before a data-driven policy and response for the region can be validly drafted.^8^ Part of the gaps that have been identified includes the observation that most of the available studies focussed on a single psychiatric disorder rather than a broad range of disorders. Focus on a single psychiatric disorder amongst a population that has been established to have multiple psychopathologies including co-morbidity is reductionist. The present study will, therefore, examine the wide range of psychiatric disorders amongst a sample of boys in a youth correctional facility in Nigeria.

Identifying the broad range of psychiatric morbidity amongst detained adolescents will help in the planning of needed mental health services and improve knowledge of how best to utilise the scarce mental health resources in such settings.

## Methods

### Study design

This was a descriptive cross-sectional study.

### Study setting

The study was carried out at the Borstal Institution, Ganmo, Ifelodun Local Government Area of Kwara State, North-Central Nigeria. It was established on 26 December 2005 by the Federal Government of Nigeria under the Nigerian Prison Service, to see to the training and reformation of delinquent youth. The institution admits two groups of residents, namely, juvenile offenders and children beyond parental control. This restrictive environment operates as a correctional facility with emphasis on educational and vocational training for residents.

Adolescents present to the institution either through their parents or law enforcement agents. The juvenile court warrant is issued for the adolescent to be admitted into the institution if found guilty of the alleged offence for a period of 6–36 months depending on the offence of the adolescent.

### Study population

The study included all detained adolescents aged 12–18 years who were incarcerated at the institution during the time of the study who agreed to participate regardless of the nature of their charges.

### Inclusion criterion

- This study included all adolescent residents of the Borstal Institution who were aged 12–18 years at the time of the study.

### Exclusion criteria

- Residents who did not consent to participate.
- Residents with severe communication disability.

### Sample size

One hundred and twenty (120) residents of the Borstal institution met the inclusion criteria and they were all recruited for the study.

### Procedure

All consenting adolescents who met inclusion criteria were included in the study. The study was carried out in two stages. In the first stage, all consenting residents who met the inclusion criteria were interviewed by a trained research assistant (Resident Doctor) using the Pro forma questionnaire and the screening aspect of Kiddies Schedule for Affective Disorders and Schizophrenia Present and Lifetime Version (K-SADS-PL)^16^ on a one-on-one basis. After the first stage, 109 Borstal institution respondents screened positive. In the second stage, all respondents who screened positive at the first stage of the interview were then each interviewed by the researcher using the diagnostic portion of K-SADS-PL. Ninety-nine (99) of the respondents were found to have psychiatric disorders. Diagnoses of psychiatric disorders were according to *Diagnostic And Statistical Manual of Mental Disorders 4th Edition* (DSM-IV) criteria.^17^

**Data analysis**

Data were analysed using EPI-INFO version 6.04 d software. Frequency tables and relevant charts to highlight important and relevant data were generated. Means and standard deviation (SD) of numeric variables were presented. Statistical significance was set at *p* < 0.05.

### Ethical considerations

Approval for the study was obtained from the Ethics and Research Committee (ERC) of the University of Ilorin Teaching Hospital (UITH). Permission and approval to the interview of the Borstal students were obtained from the Controller-General of Nigerian Prison Service, through the Controller of Prison, Kwara State Command, through the principal, borstal training institution, Ilorin, Kwara State. Permission was also obtained from the school principal, who stood in as a legal guardian, to interview the respondents. This was similar to what was performed by other Nigerian researchers because there is no explicit law on obtaining parental consent before this type of study could be performed.^17^ Laws in other parts of the world also allow the waiving of parental consent in studies of this nature because of the difficulty that may be encountered in getting parental consent.^18^ The informed assent was obtained in writing from each of the adolescents at the Borstal Institution.

## Results

### Response rate

One hundred and twenty residents met the inclusion criteria and participated in the study, making a response rate of 100%. However, 109 respondents screened positive for psychiatric disorders, of which only 99 of the respondents were found to have psychiatric disorders by the researcher.

### Socio-demographic characteristics of respondents

The age range of the respondents was between 12 and 18 years. The majority (62.5%) were above 15 years whilst the remaining were aged 12–15 years. Their mean age was 16.1 years (SD ± 1.8).

There were more Christians amongst the respondents (*n* = 65, 54.2%) and they were all actively engaged educationally. Sixteen (13.3%) of them were engaged in conventional education only, 44 (36.7%) of them were engaged in vocational education only whilst 60 (50%) of them were engaged in both forms of education (i.e. conventional and vocational).

The majority (57.5%) of the respondents were from polygamous family setting and the remaining 42.5% were from monogamous families. More Borstal respondents were raised by a single parent (19.2% mothers and 25% fathers).

Forty-three (35.8%) respondents reported abuse or neglect in the past as against 77 (64.2%) who did not report abuse or neglect.

Six (5%) of the Borstal institution respondents reported a positive history of mental illness in first degree relatives and 111 (92.5%) had no history.

### Prevalence of psychiatric disorders amongst respondents

**FIGURE 1:**
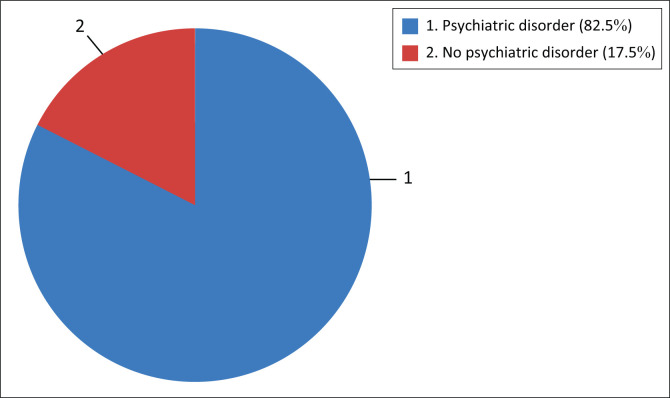
Prevalence of psychiatric disorders (*n* = 120).

The percentage of respondents with a psychiatric disorder was 82.5%, see Figure 1 for prevalence rate.

### Types of psychiatric disorders amongst respondents

The specific types of psychiatric disorders are shown (Table 2). Amongst the respondents, 30.8% had conduct disorder, 15.8% had alcohol and substance use disorder. And, 6.7% of respondents each had psychosis, post-traumatic stress disorder (PTSD) and attention-deficit hyperactivity disorder (ADHD).

**TABLE 1:** Personal characteristics of respondents.

Variables	Borstal (*n* = 120)	
	*n*	%
**Religion**		
Islam	55	45.8
Christianity	65	54.2
**Position in family**		
First born	52	43.3
Middle position	38	31.7
Last born	30	25.0
**Family type**		
Monogamous	51	42.5
Polygamous	69	57.5
**Parents marital status**		
Married	64	53.3
Separated or divorced	39	32.5
Widowed	14	11.7
Never married	3	2.5
**Care-giver**		
Father only	23	19.2
Mother only	30	25.0
Both parents	49	40.8
Relatives	18	15.0
**Occupation status of father**		
Employed	105	87.5
Not employed	15	12.5
**Occupation status of mother**		
Employed	104	86.7
Not employed	16	13.3
**History of abuse or neglect**		
Yes	43	35.8
No	77	64.2
**History of mental illness in first-degree relatives**		
Yes	6	5.0
No	111	92.5

Depression was found in 5.0%, ODD in 3.3%, phobia in 3.3%, panic disorder in 2.5% and generalised anxiety disorder in 1.7% of the respondents (see Table 2).

**TABLE 2:** Prevalence of specific psychiatric disorders amongst respondents.

Variables	Borstal (*n* = 120)	
	*N*	%
Conduct disorder	37	30.8
Alcohol and substance abuse	19	15.8
Psychosis	8	6.7
PTSD	8	6.7
Depression	6	5.0
ADHD	8	6.7
Oppositional defiant disorder	4	3.3
Generalised anxiety disorder	2	1.7
Panic disorder	3	2.5
Phobia	4	3.3
None	21	17.5

PTSD, post-traumatic stress disorder; ADHD, attention-deficit hyperactivity disorder.

When the psychiatric disorders were grouped, 40.8% of respondents had disruptive behaviour disorders (DBD), 15.8% had substance use disorders, 14.2% had anxiety disorders, 6.7% had psychosis and 5.0% had mood disorders (see Table 3).

**TABLE 3:** Prevalence of grouped psychiatric disorders amongst respondents.

Variables	Borstal (*n* = 120)	
	*N*	%
Disruptive behaviour disorders	49	40.8
Substance use disorders	19	15.8
Anxiety disorders	17	14.2
Psychosis	8	6.7
Mood disorders	6	5.0
None	21	17.5

## Discussion

### Prevalence and types of psychiatric disorders amongst respondents

#### Prevalence of psychiatric disorders

The prevalence of psychiatric disorders in this study was 82.5% for the respondents. This prevalence rate was comparable to the figures of 85.7% and 73% reported by Ulzen and Hamilton^18^ in Canada and Stahlberg et al.^12^ in Sweden, respectively, and many other studies worldwide.^12,18^

The prevalence finding of this study was, however, higher than those of Teplin et al.^15^ who found the prevalence of any psychiatric disorder of youths in the Cook County juvenile temporary detention centre in Illinois (United States of America [USA]), using the Diagnostic Interview Schedule for Children (DISC IV) to be 66.3% for males. Kenyan^14^ and Nigerian^19^ studies found lower prevalence rates of 44.4% and 67.9%, respectively. The methodological differences in these studies may partly account for the differences in prevalence rates. For instance, the study by Ajiboye et al.^19^ used a different instrument, Mini International Neuropsychiatric Interview for Children/Adolescents (MINI-KID). They had a smaller sample size and included respondents older than 18 years. The high prevalence of mental illness in this population reiterates the notion that mental illness is a justification for ill-conduct and hence incarceration.

#### Specific psychiatric disorders

The types of psychiatric disorders found in this study were conduct disorder, alcohol and substance use disorder, psychosis, PTSD, depression, ADHD, ODD and other anxiety disorders.

Disruptive behaviour disorder has consistently been found to be high in many previous studies of juveniles within the JJS.^9,10,15^ Like many other studies, this study found a high proportion of respondents with psychiatric disorders to have a prevalence rate of 40.8% for DBD. This was similar to the study by Teplin et al.^15^ who found a prevalence rate of 40% for DBD using DISC IV within a detention centre in the USA. The figure is, however, lower than a prevalence rate of 50% found in 478 youths across five sectors of care in California, the USA using computer-assisted DISC-IV.^20^ It was also lower than the prevalence rate of 75% found amongst incarcerated male adolescents in Netherlands^21^ and 67.9% in a Nigerian study.^22^ These differences could be because of the different instruments that were used, types of population studies and methodologies used. (Cultural differences between areas of study, also the combination of risk factors like low socio-economic status, single parental care and parental marital failure could also be responsible for these differences.)

Conduct disorder was the most common DBD amongst the participants. This is similar to other studies from other African countries^14,19,23^ and other parts of the world.^17,24^ The prevalence of conduct disorder in this study was similar to that obtained by Wasserman et al.^25^ amongst 292 male youths in secure facilities in the USA. McCabe et al.^26^ also found a prevalence of 33% amongst adjudicated male youths. The finding in this study is also comparable to that obtained by Garland et al.^20^ amongst 478 juvenile offenders in California.

However, the prevalence for conduct disorder obtained in this study was lower when compared with some other African studies^14,19^ that reported prevalence rates for conduct disorder of 45% and 64.2%, respectively. Bella et al.^27^ reported a prevalence rate of 18.6% for conduct problems amongst a remand population, which is much lower compared with the present study. This could be partly because of the smaller sample size of 59 participants. It is a remand population with children of younger age group, 40% were females and 90% of participants were children in need of care and protection.

It is not surprising that conduct disorder has the highest prevalence in this study. This is because for the juvenile to have been admitted to the Borstal institution, they must have had some unacceptable behaviour severe enough to make it difficult for the parents to handle. Thus, conduct disorder could be the underlying psychopathology responsible for their unacceptable behaviour leading to incarceration. Disruptive behaviour disorder which is the most common psychiatric disorder in this study was the most common reason for the inability of parents to keep their children within control necessitating placement within the JJS.

Similarly, substance use disorder has consistently been found to be high in many previous studies of children and adolescents in the JJS. This study, however, found a prevalence rate of 15.8% for substance use disorder. A study within the JJS in the US reported a higher prevalence rate of 37% for substance use disorder.^28^ This may not be unconnected to the attitude to substance use in this region. A Nigerian study reported a prevalence rate of 58% for any substance use disorder.^22^ Apart from substance abuse which could impair their judgement and increase their risk of unacceptable behaviour, these juveniles might also require money to sustain their substance use behaviour which could then make them resort to crime (e.g. stealing) and thus ultimately get incarcerated.

The present study found a prevalence of 14.2% for anxiety disorders. This comprised of PTSD, phobia, generalised anxiety disorder and panic disorder. This is similar to a previous study amongst juveniles in the JJS where the prevalence of anxiety disorder was 17%, comprising of PTSD (7.5%), panic disorder (5.7%), obsessive compulsive disorder (1.9%) and separation anxiety disorder (1.9%).^29^ Wasserman et al.^30^ also reported a prevalence rate of 20% for any anxiety disorder amongst 292 youths in secure facilities in the USA. However, a higher prevalence of 32% for anxiety disorder excluding PTSD was reported amongst adolescents in custody in Tasmania, Australia.^26^ The isolation from the social network of youths in custody may be responsible for this. Also, Garland et al.^20^ found the prevalence of anxiety disorders in California, USA to be 9% in youths across five sectors of care. This could be because of the use of different instruments, sample size and location of study. Anxiety disorder could be a function of the state of incarceration itself like sleeping in locked rooms and being separated from loved ones.

The prevalence of psychosis in this study was 6.7%. This is comparable to a Nigerian study that reported a prevalence rate of 3.8% for psychosis.^19^ A meta-analysis of 25 surveys found a prevalence rate of 3.3% and 2.7% of psychotic disorders for incarcerated adolescent boys and girls, respectively.^24^ McManus et al.^25^ reported a prevalence rate of 18% for psychotic disorders and a study of incarcerated boys in the Netherlands reported that 34% of their participants were DISC-2.3 psychosis screen positive.^21^ The psychosis module of DISC-2.3 is a broad symptom screen and does not generate a specific diagnosis, and this may have accounted for the higher prevalence in the latter study.

This study found a prevalence of 5.0% for depression. This is similar to the study by Garland and colleagues,^20^ who reported a prevalence rate for major depression of 4.7%.

Wasserman et al.^28^ reported a prevalence rate of 8% for depression amongst youths in secure facilities. The prevalence of depression found in this study was lower than 17% that was found in another Nigerian study.^22^ Also Bella et al.^27^ reported the prevalence of depressive symptoms to be 20.3%. The latter study reported depressive symptoms, not a disorder. Children in need of care and protection who were included in the latter study are more liable to depression because of the various adversities and maltreatment they may have encountered before coming in contact with the JJS.

### Limitations

It may be difficult to generalise the findings of this study because all respondents were males, which precluded the possibility of studying the contribution of gender to juvenile offending as well as to the burden of psychiatric morbidity amongst young offenders, also the sample size was small which could lead to erroneously high or low prevalence rates and the research relied on verbal self-reports from respondents only as parents, caregivers and medical records were not available to confirm some of the information.

## Conclusion

This study confirmed the high prevalence rate of psychiatric disorders amongst adolescents involved with the JJS irrespective of the type or geographical location as found in several studies worldwide including sub-Saharan Africa.

A large percentage of adolescents within the Borstal training institution had clinical psychiatric diagnoses and the pattern of psychiatric disorders included DBD, substance use disorder, anxiety disorder, psychotic disorder and mood disorder.

These findings underscore the need for policies to prevent psychiatric disorders amongst at-risk youth and to provide services for those coming in contact with the JJS. Such measures will help reduce psychiatric morbidity in adolescents, improve their quality of life and reduce the risk of reoffending. There is still a knowledge-driven consensus required on the most context-appropriate modality for providing mental health services for adolescents and youth in contact with the JJS in Africa,^31^ establishment of community-based mental health services to train correctional staff on the basic principles of mental health assessment and interventions could serve as a form of pre-occurrence diversion programs for minor and status offenders, in order to allow for capacity building within the JJS.^32,33^
